# The Role of the Clinical Pharmacist in an Irish University Teaching Hospital: A Mixed-Methods Study

**DOI:** 10.3390/pharmacy8010014

**Published:** 2020-01-30

**Authors:** Sarah Ronan, Nicola Shannon, Katie Cooke, Trish McKeon, Elaine K. Walsh, Alan Kearney, Laura J. Sahm

**Affiliations:** 1 Pharmaceutical Care Research Group, School of Pharmacy, University College Cork, Cork T12 YN60, Ireland; Sarahlouiseronan@gmail.com; 2 Pharmacy Department, South Infirmary Victoria University Hospital, Cork T12 X23H, Ireland; shannon.nicola@sivuh.ie (N.S.); katiejcooke@hotmail.com (K.C.); marypatriciamckeon@gmail.com (T.M.); 3 Department of General Practice, University College Cork T12 YN60, Ireland; Elaine.walsh@ucc.ie; 4 Pharmacy Department, University Hospital Kerry, Rathass, Tralee V92 NX94, Ireland; alankrny@yahoo.ie; 5 Pharmacy Department, Mercy University Hospital, Grenville Place, Cork T12 WE28, Ireland

**Keywords:** medication review, cost avoidance, adverse drug events, views of nurses, cost benefit ratio, semi-structured interviews

## Abstract

Medication review (MR) is a vital part of the pharmacist’s role in hospital. However, in the South Infirmary Victoria University Hospital (SIVUH), Cork, Ireland, this has not been fully implemented due to resource issues. In addition, the cost of providing this service has not been evaluated. Moreover, it is not clear how other members of the multidisciplinary team e.g., Nurses, value any interventions made as a result of the MR. This mixed methods study assessed the impact of MR in terms of (i) potential clinical harm, (ii) cost avoidance and (iii) the views of nursing staff on the role of the pharmacist. The setting is a 192-bed, voluntary, acute hospital, in the Munster region of Ireland. **Study I:** The pharmacist provided MR to patients conventionally once a week. Any interventions were then assessed for potential clinical harm and to calculate cost avoidance. **Study II:** Semi-structured interviews, guided by a topic guide were completed with 12 nurses (11 female). Thematic analysis was used to code the main themes. Main outcome measure: To estimate the cost, cost avoidance, and the net cost benefit ratio of MR provided by pharmacists. **Study I:** Of 128 patients who received the MR, 113 interventions were made. The estimated cost of providing the MR was €2559 (senior pharmacist). Using €1084 as the cost of an adverse drug event (ADE), the cost avoidance was calculated at €42,330. This led to a net cost benefit of €39,771 (senior pharmacist) which equated to a net cost benefit ratio of 16.5:1. **Study II:** The main themes were (i) perceptions of pharmacy services, (ii) the role of the pharmacist—past, present and future, and (iii) teamwork and communication. Nurses expressed a desire to have more pharmacists present on the wards.

## 1. Introduction

The traditional role of a pharmacist predominantly involved the dispensing of medications in both hospital and community settings. The profession has since evolved to become recognized as an essential part of the healthcare team [1]. Pharmacists are increasingly being viewed as key members of multidisciplinary teams in both primary and secondary care settings. A study by Makowsky et al. described how the integration of pharmacists into healthcare teams assists in improving team decision-making on drug therapy, positive patient outcomes, increasing patient safety, and improving the continuity of care [2]. A Cochrane report stated that the concept of collaboration, which is the process in which different professional groups work together, if successfully implemented, will have a positive impact on healthcare [3]. Other studies have shown that multi-professional collaboration including pharmacists, results in beneficial effects [4,5,6,7].

Clinical pharmacists are defined by the American College of Clinical Pharmacy as “practitioners who provide comprehensive medication management and related care for patients in all health care settings. They are licensed pharmacists with specialized advanced education and training who possess the clinical competencies necessary to practice in team-based, direct patient care environments [8].” A clinical pharmacy service (CPS) uses the therapeutic expertise of the pharmacist to ensure optimal patient outcomes [9,10]. Pharmacist interventions (PIs) are one of the key aspects of the new enhanced role which pharmacists offer in a hospital setting [11,12]. A pharmacist intervention (PI) is defined as “any action taken by a pharmacist that aims to change patient management or therapy [13].” This has the potential to provide an additional economic benefit to the healthcare institution, due to substantial cost avoidance [14].

Medication review (MR) is an integral part of a clinical pharmacist’s interventions. MR is undertaken to identify and reduce medication errors and to optimize the treatment of the patient [8]. MR is defined as “a structured, critical examination of a patient’s medicines with the objective of reaching an agreement with the patient about treatment, optimizing the impact of medicines, minimizing the number of medication-related problems and reducing waste” [15]. Increasing drug consumption together with patient metabolic changes with age predisposes older people to medication related problems e.g., reduced elimination due to renal impairment, and ADEs [16,17]. Medication reconciliation is the process of creating the most accurate list possible of all medications a patient is taking, including drug name, dosage, frequency, and route, and comparing that list against the physician’s admission, transfer, and/or discharge orders, with the goal of providing correct medications to the patient at all transition points within the hospital [18]. Previous studies have shown the importance of undertaking MR as a means of identifying medication-related problems and thus positively contributing to patient care [19,20,21,22].

The South Infirmary Victoria University Hospital (SIVUH) is an acute urban university teaching hospital located in the city of Cork, in the South of Ireland. The SIVUH has a total designated bed complement of 192 beds and caters for up to 38,400 admissions and 72,500 outpatient attendances each year. At the time of the study, there were four full time and one part time pharmacist employed. There is currently no ward-based pharmacy service but rather one which focuses on supply of medication and answering medication queries from nursing and medical personnel over the telephone. While some pharmacists undertake MR, this is not always on a regular basis, which means that a business case needs to be made to justify the redeployment of a pharmacist from the dispensary to the ward. It also means that nurses may not be fully aware of the skillset of pharmacists and the support that they can offer on the ward. Nurses are involved in the ordering, storage, and administration of medication to patients on the wards and are the person most likely to have to resolve medication related queries, in the first instance. 

Gillespie et al. examined the experiences of physicians and nurses involved in a randomised controlled trial of ward-based clinical pharmacists at two internal medicine wards at Uppsala University Hospital [23,24]. This descriptive study demonstrated that clinical pharmacists’ collaboration with physicians and nurses in hospital increased patient safety and improved the quality of prescribing and that doctors perceived this collaboration as beneficial both for themselves and their patients [23]. In a study, from South Korea, the nurses interviewed discussed the need for pharmacists in improving communication, drug counselling, providing information about drug distribution in the market, implementing an easy-to-use drug identification system, providing a medication reconciliation service and providing education to nurses about medications [25]. In the study by Salgado et al., nurses from Australia and Portugal were interviewed and whilst Australian nurses were aware of the clinical competencies of pharmacists, in contrast, Portuguese nurses appeared to have little knowledge of pharmacists competencies [26]. A further Swedish study evaluated the views of hospital personnel prior to the introduction of a CPS. Unsurprisingly the results showed that nurses had limited knowledge of the pharmacists’ role on the ward. They described traditional roles of pharmacists such as inventory and drug distribution [27]. The participants reported uncertainty about the ward-based clinical pharmacist role and unclear about their clinical skills and competencies [27]. 

The aims of this study were to assess the impact of a MR service by a pharmacist and then explore the views of nursing staff on the role of the pharmacist in the SIVUH. 

**Objectives:**

**Study I:**

1. To identify the types and prevalence of PIs and the medication most frequently implicated.
2. To measure PI acceptance and implementation rate by physicians.
3. To determine the probability that an adverse drug event (ADE) would have occurred if the PI had not taken place, using the methodology utilised by Gallagher et al., which was adapted from Nesbit et al. [14,28].
4. To evaluate the cost-effectiveness of the MR and to calculate the net cost benefit and the cost benefit ratio.

**Study II:**

After completion of Study I; to interview SIVUH nursing staff to explore their views on the pharmacy services.

## 2. Materials and Methods 

**Study I:** The clinical pharmacist (SR) provided MR to patients of the four study wards (n = 74 beds) conventionally once a week over a three-month period from March–May 2018. These wards were chosen to represent a mixture of male/female and medical/surgical wards. Inclusion criteria of patients: (i) age over 18 years (ii) drug kardex available for review. Exclusion criteria were patients on specialty wards, e.g., oncology, as they receive chemotherapy using a specific prescription form. The MR was standardized: Every medication review included medication reconciliation. Sources of information included patient’s medical notes, GP (written/verbal) and patient’s community pharmacist (written/verbal) in order to assess the patient’s drug kardex. The drug kardex is a hardcopy A4 booklet, normally located at the end of a patient’s bed and contains a list of the patient’s medication since admission to hospital. Each medication was checked for clinical accuracy e.g., in terms of drug indication, dose, route, frequency and duration. These checks were supported by the patient’s medical notes, which detail the list of medication upon admission, as well as laboratory data, specifically to monitor any “out of normal limits” results on electrolytes and renal and hepatic function amongst others. Patients were also be consulted regarding their medication, where necessary. 

When an issue was identified, a note was written in pen on a Pharmacy notepad, which had a carbon copy, detailing the PI and recommendations from the pharmacist, and this was attached to the drug kardex for review by the Medical/Surgical team. As the team generally sees patients once daily, rather than wait for the following day, the pharmacist contacted, via phone/bleep a member of the surgical or medical team, responsible for the patient and verbally informed this member (usually a Non- Consultant Hospital Doctor (NCHD)) of the PI(s). Where possible, the PIs were communicated face-to-face. The carbon copy was retained by the pharmacist for data collection and follow-up. PI acceptance was determined from the outcome of the conversation with the patient’s medical/surgical team, i.e., if the member of the team agreed with the recommendation and gave an undertaking to make the change suggested by the pharmacist. PI implementation was determined by the pharmacist reviewing the drug kardex and checking whether this change had been made (between 24 and 72 h). If the change had not been implemented within 72 h, it was scored as “not implemented”. The PIs were classified based on the system used by Kearney et al. [21]; based on the system used by Gallagher et al. [7]. One addition, “errors in taking of medication history”, was added. 

### 2.1. Assessment of Potential Clinical Harm

PIs were allocated a probability score which indicated the likelihood that an ADE would have occurred, in the absence of the MR. This was based on previous studies [7,14]. Three clinical pharmacists, an academic pharmacist and a general practitioner (GP) rated the PIs. The median probability score for each of the PIs was used for analysis. An inter-rater reliability (IRR) analysis using the Kappa statistic determined consistency amongst the raters [29]. (Table 1) 

The following information was given to assessors as an aid to determine the probability of an ADE occurring. (Table 2).

The cost was calculated based on the hourly cost of a basic grade pharmacist (€38.15/hour and annual salary of €45,247) and a senior pharmacist (€55.33/hour and annual salary €65,628) at the midpoint of the HSE 2018 salary scale [30]. These values were revised to include pay-related social insurance (PRSI), pension contribution and general overheads that occur within the Irish healthcare system [31].

Cost avoidance was assessed based on the likelihood that an ADE would have occurred in the absence of the PI. The cost of an ADE used was €1084 taken from previous literature [14] and adjusted for inflation [32]. The cost avoidance was calculated by multiplying the cost of an ADE (€1,084) by the median probability score given to the PI by the raters. The total cost avoidance was calculated by adding the cost avoidance of the individual PIs. The operating cost of MR by pharmacists and the cost avoidance were used to calculate the net cost benefit (cost avoidance minus the cost of service). The operating cost of MR by pharmacists and the cost avoidance were also used to calculate the cost benefit ratio (cost avoidance divided by cost of service).

### 2.2. Data Analysis

Descriptive results were generated using Microsoft Excel 2010 and IBM Corporation Statistical Package for the Social Sciences (SPSS) Version 24 was used for statistical analysis (with an a priori level of statistical significance set at *p* < 0.05). Ethical approval for was granted by the Clinical Research Ethics Committee of the Cork Teaching Hospitals, University College Cork (UCC), Cork, Ireland and from the SIVUH Board of Directors. 

**Study II:** Convenience sampling was used. The researcher approached nurses who were available and likely to participate. Snowball sampling was also used, based on referrals from participating nurses. We aimed to conduct interviews until data saturation had occurred and no new themes emerged and thus a preliminary sample size of 10 interviews as per Francis et al. was decided upon [33]. Inclusion criteria were that the nurse was currently employed on one of the four study wards and had been employed there for over six months. Exclusion criterion: those working as agency staff. 

All participating nurses provided written informed consent. Participants were assured that all information given was confidential. Information on nurse demographics was also gathered. Interviews were semi-structured and guided by a topic guide (Appendix A). The topic guide was developed based on the researcher’s own experience and a preliminary review of the literature. Interviews were conducted until no new themes emerged. Interviews were recorded using a digital voice recorder and transcribed verbatim. All transcripts were read several times, coded and analysed for recurrent themes. Thematic analysis was employed [19]. The interview transcripts were reviewed and coded independently by the academic supervisor to ensure rigour. Ethical approval was granted by the Social Research Ethics Committee of the Cork Teaching Hospitals, UCC, Cork, Ireland and the SIVUH Director of Nursing.

## 3. Results

**Study I:** An estimated 1135 patients were inpatients on the study wards [34] for the duration of the study. A total of 128 patients of 142 eligible, received a MR and this equated to 46.25 h. A total of 113 PIs were identified in 59 patients. The demographics of patients with PIs are shown in Table 3.

This represents a mean of 1.92 PIs per patient who had a PI. A mean of 0.88 PIs was found per patient who received a MR. The most common intervention noted was that of medication omission. See Table 4 for the type and prevalence of PIs.

Analgesics are the most common medicine type associated with PIs. Oxycodone and paracetamol were the most frequently associated, with each accounting for five PIs. Psychoanaleptics that were associated with multiple PIs included amitriptyline, citalopram and mirtazapine. Regarding anti-inflammatory drugs, dexketoprofen was most commonly associated with PIs (seven PIs) (Table 5).

### 3.1. Acceptance/Implementation of PIs

Of the 113 PIs, 112 (99.1%) were accepted by the patient’s medical/surgical team and of these, 100 (88.5%) were implemented. The PIs were allocated a probability score indicating the likelihood that an ADE would have occurred if the PI had not taken place. Examples of PIs and their allocated median probability scores, as assigned by the raters, are shown in Table 6.

According to this score, 66.4% of the PIs had a medium or high likelihood of causing an ADE, using the median value assigned by the PI raters. An IRR analysis using the Kappa statistic was performed to determine absolute agreement among raters, which ranged from 0.003–0.623 [15].

### 3.2. Cost Analysis

The cost of providing the MR was estimated using the hourly rate of a basic grade pharmacist at the midpoint of the salary scale. The total cost for providing the service to the four wards during the three-month study period was €1764 based on 46.25 h delivering the MR and an hourly rate of €38.15. The cost avoidance for each PI was calculated by multiplying the cost of an ADE (€1084) by the median probability score given to the PI by the raters. The total cost avoidance was calculated by adding the cost avoidance of the individual PIs. The cost avoidance associated with providing the MR during the study period was €42,330 based on the likelihood that an ADE would have occurred in the absence of the PI and the cost of an ADE of €1084. The theoretical operating cost of implementing the MR and the cost avoidance were used to calculate the net cost benefit (cost avoidance minus the cost of service) and was €40,566. The operating cost of MR and the cost avoidance were also used to calculate the cost benefit ratio (cost avoidance divided by cost of service) which was 23.99:1. 

For a senior pharmacist, the total cost for providing the service to the four wards during the three-month study period was €2,559 based on an hourly rate of €55.33. The cost avoidance associated with providing the MR during the study period was €42,330 based on the likelihood that an ADE would have occurred in the absence of the PI and the cost of an ADE of €1084 and the net cost benefit was €39,771. The cost benefit ratio was 16.54:1.

**Study II:**

Twelve nurses involved in the care of patients in the SIVUH agreed to participate. Of the nurses interviewed, eleven were female, and were employed on a full-time basis. Ten of the nurses were aged between 20 and 39. The level of experience varied, six of the nurses had worked for 0–9 years in practice and six had worked for over ten years. Three main themes were identified from the interviews.

1. Perceptions of pharmacy services
2. The role of the pharmacist —— past, present and future
3. Teamwork and communication

### 3.3. Perceptions of Pharmacy Services

Overall, the nurses expressed satisfaction with the current service and the perceptions of the Pharmacy Department were positive.

*“Yeah, I wouldn’t have any ideas now to improve it.”*

(Nurse 3)

Positive opinions were expressed on recent quality improvement incentives from the Pharmacy Department, such as improvements to the drug kardex.

*“No as I said, I didn’t like the old kardex so I like the new one, you’ve changed that. You seem to be doing a lot of audits on, you know, our record-keeping and medications so that kind of, it’s another incentive to keep us on our toes, which I think helps.”*

(Nurse 6)

However, some nurses showed negative attitudes towards the current pharmacy services. Negative views relating to lack of a ward-based MR and the micromanagement of drug quantities were expressed.

*“Because I came from the X hospital and routinely the pharmacists would come down every day more or less and review drug kardexes. I find here you are at a disadvantage because that’s not routinely done. And it’s kind of up to you as a nurse to kind of notice the discrepancies... Yeah, it’s a pity I think. Because we are still quite acute, I think there could be more pharmacy presence here….”*

(Nurse 1)

*“I don’t like the way as well here, everything is under the thumb like. A patient could be in for two days, but like you get two aspirins. Sure that’s ridiculous. It’s micro managing.”*

(Nurse 1)

### 3.4. The Role of the Pharmacist——Past, Present and Future

All the interviewees described the current role of the pharmacist in the SIVUH as the traditional role of the pharmacist.

*“Taking requisitions from every ward, dispensing the medicines, keeping an eye on ward stock down here on the wards.”*

(Nurse 8)

Many nurses saw a future role for pharmacists in the area of medication reconciliation and MR.

*“Yeah, they could do more I think. They do help but like we don’t ring them if there is an admission, we do it ourselves and it happened a few times that we would pick up on something and then we ring up and then the (SIVUH) pharmacist would say I will just ring the pharmacist, the patient’s pharmacist, to sort it out and that’s really good like.”*

(Nurse 5)

MR was an activity that nurses viewed as beneficial and many nurses valued the double-checking of the drug kardex by pharmacists.

*“I suppose like I love coming to see a drug chart and the green pen. I love seeing that. So at least you know then that everything is double-checked and the pharmacist always explains things a little bit more.”*

(Nurse 3)

It was evident from the interviews that nurses valued the medication reviews (MRs) completed. Some nurses perceived it to minimise errors and focus attention on good prescribing practices.

*“I think the situation at the moment, with the audits (medication reviews completed as part of study one) is good because I do find myself double-checking the kardex to see if everything is right, which obviously helps eliminate errors and all that kind of thing so. Whereas when you’re not around I find people are just a bit more blasé about it like.”*

(Nurse 6)

Some nurses emphasised that the continuation of the MRs (completed for **study I**) would be beneficial.

*“No, I think it would be better if there was more. Sometimes, recently they’ve been coming down and doing the drug charts, that’s really good so if they could do that all the time, that would be way better.”*

(Nurse 5)

### 3.5. Teamwork and Communication

The involvement of the pharmacist on the multidisciplinary team was discussed but there is a need for greater involvement in the multidisciplinary team.

*“I suppose maybe a mixture of the multidisciplinary team, like there’s us, there’s the doctors, there is ye. It would be great if we could get an input from everybody.”*

(Nurse 7)

Nurses expressed positive opinions on the support provided to them by pharmacists. Working relationships appeared strong and nurses viewed pharmacists as approachable. Nurses viewed pharmacy as a consistent support that had undergone improvements in recent times.

*“You are always there to support us, I must say that, you are always there and I mean, certainly things have improved in the last year.”*

(Nurse 9)

## 4. Discussion

This is a mixed methods study in an acute university teaching hospital which sought to combine the results of a quantitative estimate of the benefits of having a clinical pharmacy service, with the views of key stakeholders on the wards i.e., nursing staff. The number of PIs found per patient who received a MR in this study was 0.88. This was within the range of 0.13 [22] to 9.35 [37] reported in the review of the literature [19,21,22,37,38,39,40,41,42]. The reported rate of this study is towards the lower part of the range. This could be attributed to the time constraints of study one, whereby the study wards visited once a week where possible. 

Omissions, errors in taking medication history and duplication were the most commonly encountered PIs, representing almost two in every three of the PIs identified. Other studies have found omission of drug to be one of the most commonly encountered PIs [14,38]. All of the omissions are attributable to lack of accurate medication [14,41,43] reconciliation on admission to or while in hospital. Omissions of medication may have potentially serious consequences for patients depending on the nature of the drug omitted [44]. “Errors in taking medication history” was a PI category added by the researcher. In the SIVUH, medication reconciliation on admission is not routinely carried out by a pharmacist. A lack of medication reconciliation at admission and discharge from acute hospital care is common and has the potential to cause patient harm [44,45,46]. 

In Ireland, the frequency and consistency of delivering pharmacy services to facilitate medication reconciliation at admission and discharge could be improved [47]. A study by Grimes et al. of acute, public hospitals in Ireland found all hospitals surveyed provided acute care and by Australian standards (recommended 30–40 beds per clinical pharmacist), they were poorly resourced to deliver a MR to patients of that complexity [47]. Grimes et al. found a mean of 70.2 beds per whole-time equivalent (WTE) pharmacist in similar-sized Irish hospitals to SIVUH [47]. Currently in the SIVUH, there is one WTE pharmacist allocated to inpatient clinical services, in a 192-bed hospital. It would therefore be considered poorly resourced by both national and international standards. Solutions to this could include the upskilling of pharmacy technicians to take on medication reconciliation particularly in environments where resources are scarce [48]. The advantage would be that this could be done at a lower cost, as technicians are paid less, but this would however require training, support and supervision from a clinical pharmacist to support the technician and ensure that they are competent. An alternative option would be to prioritize patients based upon their likelihood of suffering an ADE, in line with risk factors such as age, gender and co-morbidities. These are options that should be considered if the current situation persists. 

Analgesics were the most common medicine type associated with PIs in this study. Previous work has reported analgesics amongst the most common medicine type associated with PIs [22,43]. Analgesic drugs such as paracetamol and oxycodone were the most frequently duplicated in this study also. Duplication often occurs when paracetamol and oxycodone are prescribed as a regular and “as required” medicine in error whereby the cumulative dose exceeds the intended dose or as in the case of paracetamol the maximum daily licensed dose [35]. Annual reports from the National Poisons Information Centre (NPIC) in Ireland and the National Poisons Information Service (NPIS) in the UK identify paracetamol as the most common drug involved in human poisoning [49,50]. 

A potentially serious duplication of the anticoagulant drug class occurred when tinzaparin and rivaroxaban were co-prescribed. Direct oral anticoagulants (DOACs) are high-risk drugs, with risks including bleeding or haemorrhage, particularly when prescribed in combination with medicines increasing the bleeding risk such as low molecular weight heparins (LMWH) [51]. 

### 4.1. PI acceptance and Implementation

Almost all (99.1%) of the PIs were accepted by the patient’s medical/surgical team. This acceptance rate was within the range of studies in the literature. The range was from 59.9% to 100% [22,41]. This high acceptance rate reflects the clinical relevance of the PIs [52]. All of the PIs were communicated by direct contact via telephone/bleep, in many instances this was actually face-to-face contact if possible. This agrees with studies that have shown a significant positive association between direct oral communication and PI acceptance by physicians [21,37,40].

The ultimate objective is for the recommended change to be implemented after being accepted. 88.5% of the PIs were implemented by the patient’s team. Implementation rates were shown to be lower than the acceptance rates of PIs in the studies of AbuRuz et al. and Movva et al. [37,39].

The high implementation rate of this study can be attributed to the supply of adequate information with the PI(s), the prompt and face to face communication and the significance of the PI(s). This rate could be made higher by incorporating a pharmacist as part of the multidisciplinary team to improve medicines optimization [53]. 

### 4.2. Cost Analysis

This study found significant cost benefits and the cost benefit ratio was 16.54:1 for a senior pharmacist and 23.99:1 for a basic grade pharmacist. A similar study by Gallagher et al. reported a cost benefit ratio of 8.64:1 [14]. Another similar study in Ireland, found that the MR provided resulted in a cost benefit ratio of 13.66:1 [54]. In this study, 66.37% of the PIs had a medium or high likelihood of causing an ADE, which is higher than the other similar studies, which had values of 28.7% [14] and 60.7% [54]. Whilst it could be argued that the Kearney et al study yielded similar results [54], the Gallagher et al. study is a lot lower [14]. Upon further inspection however, this study was based upon a random sample of interventions (n = 100) which were then reviewed by two academic pharmacists with hospital pharmacy experience, rather than by five raters, including a GP, as in our study [14]. This measurement of potential clinical harm of the PIs was used to calculate the cost avoidance, which was then used to calculate the net cost benefit and cost benefit ratio. Therefore, the higher ratings of potential clinical harm in this study result in a higher cost benefit ratio.

**Study II:**

### 4.3. Perceptions of Pharmacy Services

To date in Ireland, there has been little published research conducted to explore the views of nursing staff on the role of the clinical pharmacist. Overall, in this study, nurses had a positive perception of pharmacists. The nurses interviewed expressed opinions of pharmacists in a proactive sense, detailing pharmacy initiatives such as the development of a new drug kardex and procurement of individualized patient medication lockers. Positive perceptions were also found in the literature [9,11,12]. On the other hand, some nurses perceived the pharmacists as reactive and involved in micromanagement. There were similar findings of negative opinions in the literature review [9,10,12]. 

### 4.4. The role of the Pharmacist–Past, Present and Future

Currently in the SIVUH the MR is limited to high-risk areas. Pharmacy staffing levels do not allow for further ward-based clinical services. The Health Information and Quality Authority (HIQA), an independent authority that exists to improve health and social care services in Ireland, noted the absence of a ward-based MR at the inspection of medication safety in August 2017 [42]. 

The perception of nurses that there is a lack of MR on the wards is an agreement with the findings of the HIQA report. However, in some cases, perceptions of pharmacy services were different to reality. Whilst the nurses interviewed showed good knowledge of the traditional role of the pharmacist, sometimes they were not aware of some of the roles undertaken by pharmacists.

The role of the pharmacist was perceived in a traditional sense also in the study by Sjolander et al. [12]. Sjolander et al. found that nurses had limited knowledge of the pharmacists’ role on the ward [12]. They described traditional roles of pharmacists such as inventory and drug distribution [12].

There was a view that the pharmacist was underutilized. Nurses felt that pharmacists could be involved in the areas of medication counselling, medication reconciliation and MR.

Whilst study I found that 44.25% of PIs were due to omission of drugs and errors in taking medication history and showed that this could be improved by the implementation of a MR involving medication reconciliation. Study II found that most nurses wanted more pharmacist involvement in medication reconciliation. This viewpoint was mirrored in the literature [9,10,11].

The nurses highly valued the pharmacist-led MR as it reassured the nurses that the patient was receiving appropriate, safe and effective medicines. The perceived benefits of pharmacist-led MR were reported in the literature [9,11]. In the study by Gillespie et al. the majority of the nurses were very satisfied with the new collaboration with clinical pharmacists, which involved MR [9]. Salgado et al. found that Australian nurses would value pharmacist-led MR in the future [11]. Whilst study one showed the economic and safety benefits of the provision of a pharmacist-led MR, study two highlighted that this service was greatly valued by nurses. Nurses noted more pharmacist presence on the wards as the researcher undertook MR as part of study I and requested that this be continued in the future due to the perceived benefits.

### 4.5. Teamwork and Communication

The importance of teamwork and communication was an evident theme, there was a view that the pharmacist was a valuable team member but could be more involved in the multidisciplinary team. Gillespie et al. discussed how clinical pharmacists can collaborate with physicians and nurses in the healthcare team to improve quality of prescribing and increase patient safety [9]. This concept of collaboration and teamwork was also evident in the interviews with the SIVUH nurses. Lee et al. found that for successful collaboration of pharmacists with other healthcare professionals, efficient communication and inter-professional trust are key [10]. The SIVUH nurses believed that there was good communication and trust between the professions.

This study adds to the literature by investigating the views of other healthcare professionals, in this case nurses, after the implementation of medication reviews by a pharmacist. However, it is not without its limitations, which include a single site, a small sample size and the fact that we have only calculated the operating cost of the MR, rather than the implementation cost, the latter of which will necessarily include hardware, software, and training amongst others. 

## 5. Conclusions

This study found a prevalence of 0.88 PIs per patient who received a MR by a pharmacist, in a hospital setting in Ireland. The findings of this study add to the current evidence that MR plays a vital role in preventing potential ADEs and generating cost savings. The results of this study demonstrate the cost effectiveness of the provision of a pharmacist-led MR to inpatients. 

Perceptions of pharmacy services varied amongst the nurses interviewed. Overall, satisfaction with pharmacy services was expressed. Nurses who had previous experience of working with pharmacists on hospital wards expressed the desire to have this put into place. It is hoped that as a result of this study this demand for a more proactive pharmacy service, including MR, will be met. 

This study highlights the importance of pharmacists in the provision of timely information to the medical and surgical teams as a way of optimizing medication.

## Figures and Tables

**Table 1:** Interpretation of Kappa measure of agreement [25,29].

Kappa Measure of Agreement	Interpretation
<0	Poor agreement
0.0–0.20	Slight agreement
0.21–0.40	Fair agreement
0.41–0.60	Moderate agreement
0.61–0.80	Substantial agreement
0.81–1.00	Almost perfect agreement

**Table 2:** Probability scores for assessment of potential clinical harm of the PIs provided to the raters.

Probability of an ADE Occurring	Probability Score	Example
No harm expected	0	Pharmacist suggest changing a patient from esomeprazole to omeprazole for economic reasons
Very low	0.01	Patient regularly takes a bisphosphonate, but medication omitted from drug kardex
Low	0.1	Patient takes an antibiotic twice a day, when the recommended dose would be three times a day
Medium	0.4	Metformin dose not reduced despite patient demonstrating renal impairment
High	0.6	Patient prescribed amiodarone while taking digoxin without any reduction in digoxin dose

**Table 3:** Demographics of patients with PIs.

Demographic	Description	Patients with PI(s) n = 59
Gender (n)	Male	36 (61%)
	Female	23 (39%)
Specialty (n)	Medicine	3 (5.1%)
	Surgery	56 (94.9%)
Age (years)	Median	70
	Interquartile range	23
Regular medicines	Median	11
	IQR	6
PRN medicines	Median	4
	IQR	3

Abbreviations: PI(s): pharmacist intervention(s); n: number of patients; IQR: interquartile range; PRN: “as required”.

**Table 4:** Types and prevalence of PIs.

Type of PI	No. of PIs (%)
**Omission ^1^**	**32 (28.3%)**
**Errors in taking medication history ^1^:**	**18 (15.9%)**
Incorrect frequency	11 (9.7%)
Incorrect dose	4 (3.5%)
Incorrect formulation	1 (0.9%)
Incorrect drug and dose	1 (0.9%)
Incorrect drug and strength	1 (0.9%)
**Duplication:**	**18 (15.9%)**
Co-prescribe same drug	10 (8.6%)
Co-prescribe same drug class	8 (7.1%)
**Poor prescribing practice:**	**17 (15.0%)**
Frequency of administration unclear	7 (6.2%)
Dose charted unclear	6 (5.3%)
Drug charted unclear	4 (3.5%)
**Dose ^2^:**	**8 (7.1%)**
More than licensed dose	6 (5.3%)
Less than licensed dose	2 (1.8%)
**Other:**	**8 (7.1%)**
**Interaction ^2+3+4^**	**6 (5.3%)**
Pharmacokinetic	3 (2.7%)
Pharmacodynamic	3 (2.7%)
**Timing ^2+3^**	**3 (2.7%)**
**Frequency ^2^**	**2 (1.8%)**
Less than licensed frequency	2 (1.8%)
**Duration**	**1 (0.9%)**

Abbreviations: PI: pharmacist intervention; PIs: pharmacist interventions. ^1^ Drug kardex was checked for errors using admission notes and/or contacting community pharmacy and/or contacting general practitioner. ^2^ As per Summary of Product Characteristics. ^3^ As per British National Formulary 75 [35]. ^4^ As per Lexicomp^®^ Drug Interactions checker [36]. Terms in bold are the main headings, and underneath are specific examples.

**Table 5:** Types and prevalence of medicines associated with PIs.

ATC Code	Medicine Type	Frequency Identified ^1^ (%)
N02	Analgesics	17 (11.2%)
N06	Psychoanaleptics	15 (9.9%)
M01	Anti-inflammatory and anti-rheumatic products	13 (8.6%)
A11	Vitamins	13 (8.6%)
R03	Drugs for obstructive airway diseases	12 (7.9%)
N05	Psycholeptics	9 (5.9%)
A12	Mineral supplements	8 (5.3%)
C10	Lipid modifying agents	6 (4.0%)
C03	Diuretics	5 (3.3%)
Others	Others	54 (35.5%)

Abbreviation: ATC: Anatomical Therapeutic Chemical Classification System. ^1^ Some of the interventions involved multiple medicines, for example duplication of drugs within the same drug class would be counted as two medicines.

**Table 6:** Examples of PIs and assigned median probability scores.

Probability of an ADE Occurring	Median Probability Score	Example
No harm expected	0	Medication reconciliation completed. Omission of Decavit plus^®^ tablets (multivitamin) on kardex.
Very low	0.01	Duplication of Movicol^®^ (macrogol) sachets on kardex.
Low	0.1	Durogesic^®^ (fentanyl) patch 50 micrograms/72 h prescribed. Error in calculation of timing of application of patch on kardex and the patch was applied one day late.
Medium	0.4	Overdose of Keral^®^ (dexketoprofen), prescribed orally 50 mg three times daily, maximum daily oral dose is 75 mg a day i.e., double the maximum daily dose was prescribed.
High	0.6	Xarelto^®^ (rivaroxaban) and Innohep^®^ (tinzaparin) prescribed at the same time on kardex.

Abbreviation: ADE: Adverse drug event.

## Appendix A: Topic Guide for the qualitative interviews:

- What is your gender, age, number of years in practice and are you employed on a full-time or a part-time basis?
- What do you know about the Pharmacy Department in SIVUH?
- What is the role of the pharmacist in SIVUH?
- What is your view on pharmacist presence on the wards?
- In what situations would you contact the Pharmacy Department?
- What is your view on the online referral form to request a pharmacist’s review?
- What is your view on the online referral form to request patient education/counselling on new medications?
- Who do you feel is responsible for counselling patients on new medications/changes in medications?
- How do you conduct medication reconciliation?
- How could we improve pharmacy services at SIVUH?
- Are there any additional comments that you would like to make?
